# Serodiagnosis of Human Strongyloidiasis in Brazil and Peru Using *Strongyloides venezuelensis* Antigens: A Comparative Study in Endemic Settings

**DOI:** 10.1111/pim.70109

**Published:** 2026-10-21

**Authors:** William Henry Roldán, Marcelo Andreetta Corral, Dirce Mary Correia Lima Meisel, Marco Canales, Maria Angelica Terashima, Silvia Vanessa Lourenço, Ronaldo Cesar Borges Gryschek, Fabiana Martins de Paula

**Affiliations:** ^1^ Laboratório de Investigação Médica – LIM/06 (Imunopatologia da Esquistossomose) do Hospital das Clínicas da Faculdade de Medicina da Universidade de São Paulo São Paulo Brazil; ^2^ Laboratorio de Parasitología, Instituto de Medicina Tropical “Alexander Von Humboldt”, Universidad Peruana Cayetano Heredia Lima Perú

**Keywords:** antibody, antigen, Brazil, Peru, serodiagnosis, *Strongyloides venezuelensis*, strongyloidiasis, tropical neglected disease

## Abstract

Strongyloidiasis is a neglected tropical disease caused by the soil‐transmitted helminth *Strongyloides stercoralis*. Immunological assays employing heterologous antigens from *Strongyloides venezuelensis* represent a valuable alternative to parasitological diagnosis. In this study, we evaluated the diagnostic performance of different antigenic preparations derived from *S. venezuelensis* infective larvae (L3) for the detection of anti‐*Strongyloides* IgG antibodies in sera from Brazilian and Peruvian individuals. Total soluble (S‐L3), membrane (M‐L3), cuticular (C‐L3) and excretory/secretory (ES‐L3) antigenic extracts were assessed using enzyme‐linked immunosorbent assay (ELISA). Overall, Brazilian sera showed higher reactivity than Peruvian sera to all antigenic preparations. Both S‐iL3 and ES‐L3 demonstrated excellent diagnostic performance with samples from both countries, showing 100% sensitivity and specificities of 97.4% and 100%, respectively. When a single cut‐off value applicable to both populations was adopted, S‐L3 showed the best overall performance, achieving 100% sensitivity and 98.7% specificity at a cut‐off of 0.367, or 97.5% sensitivity and 100% specificity at a cut‐off of 0.416, followed by ES‐L3, C‐L3 and M‐L3. Heterologous antigenic extracts from *S. venezuelensis*, particularly S‐L3, may represent a useful alternative for the serological diagnosis of *S. stercoralis* infection in endemic settings such as Brazil and Peru.

## 1 Background

Strongyloidiasis is a parasitic disease mainly caused by the soil‐transmitted helminth *Strongyloides stercoralis*, and it is estimated that around 600 million people are infected worldwide [1, 2]. In 2021, human strongyloidiasis was included in the group of Neglected Tropical Diseases by the World Health Organization [3].

In general, patients infected with *S. stercoralis* suffer from mild symptoms, but some progress to severe forms of hyperinfection and disseminated disease (usually seen in immunosuppressed patients), resulting in high mortality rates [4, 5]. Assessing the real impact of strongyloidiasis on human health remains a challenge probably due to the complexity of the parasite's biological cycle, the absence of specific symptoms in infected individuals and the low sensitivity of available laboratory diagnostic methods [3].

Definitive diagnosis of *S. stercoralis* infection is challenging due to the absence of a reference test to detect the parasite with high sensitivity and specificity [6]. Immunological techniques based on the detection of antibodies against the parasite have been considered a relevant detection alternative, since they can overcome the low sensitivity of traditional parasitological tests [7, 8]. Nevertheless, the main limitation in the serodiagnosis of strongyloidiasis is the difficulty in obtaining *S. stercoralis* antigens [9]. Several authors have demonstrated that the rodent parasitic nematode *S. venezuelensis* represents an excellent alternative source of antigens, due to its antigenic similarity with *S. stercoralis* and its easy maintenance in laboratory animals [9, 10]. Over the years, our group has obtained consistent and promising results using different *S. venezuelensis* antigenic preparations in serum samples from diverse Brazilian populations [11, 12, 13, 14].

Given the endemicity of human strongyloidiasis in tropical and subtropical regions, mainly in South America and the limited availability of specific epidemiological data [15, 16, 17], a study focused on immunoserological recognition across distinct epidemiological groups is timely and highly relevant. Thus, this study aimed to compare the performance of different antigenic preparations of *S. venezuelensis* in detecting anti‐*Strongyloides* IgG antibodies in human serum samples from Peru and Brazil.

## 2 Methods

### 2.1 Maintenance of the *S. venezuelensis* Life Cycle and Production of Infective Larvae

The *S. venezuelensis* strain was maintained through serial passages in 30‐day‐old male Wistar rats ( *Rattus norvegicus* ). Briefly, the rats were experimentally infected subcutaneously with approximately 20,000 infective larvae of *S. venezuelensis* (L3) obtained from 72‐h charcoal‐faecal cultures of infected rats as described previously [13].

### 2.2 Soluble and Membrane L3 Extracts

Soluble and membrane L3 extracts were obtained following the procedure of Corral et al. [11]. Briefly, approximately 200,000 L3 were resuspended in 1 mL of 25 mM Tris–HCl, pH 7.5, containing complete Protease Inhibitor Cocktail (Sigma‐Aldrich Chemical Co., Saint Louis, MO, USA), and sonicated on ice using five cycles of 20 s pulse each. The mixture was centrifuged at 12,400*g* for 30 min at 4°C and the supernatant yielded the soluble L3 extract (S‐L3). The pellet was resuspended in 25 mM Tris–HCl, pH 7.5, 7 M Urea, 2 M Thiourea and 2% CHAPS detergent and homogenised on ice for 30 min; the mixture was centrifuged at 12,400*g* for 30 min at 4°C and the supernatant yielded the membrane detergent iL3 extract (M‐L3). Both antigenic extracts were stored at −20°C until use.

### 2.3 L3 Surface Extract

Surface extract from *S. venezuelensis* L3 (C‐L3) was obtained according to the procedure described by Gomes et al. [12]. Briefly, 200 mg (wet weight) of frozen L3 were resuspended in 1 mL of buffered phosphate saline, pH 7.2 (PBS) containing 0.25% of the cationic detergent CTAB (cetyltrimethylammonium bromide) and incubated for 2 h at 4°C. The mixture was centrifuged at 10,000*g* for 10 min, and the supernatant was collected and stored at −20°C until use.

### 2.4 Excretory/Secretory Products From L3

E/S products from L3 (ES‐L3) were obtained according to Roldán et al. [13]. Briefly, approximately 400,000 live iL3 were placed in polypropylene tubes containing 5 mL of RPMI 1640 culture medium supplemented with a mixture of antibiotics (penicillin 100 U/mL, streptomycin 100 U/mL, gentamicin 50 μg/mL and amphotericin 2.5 μg/mL) and incubated for 48 h at 37°C under 5% CO_2_. The supernatant was collected and mixed with complete Protease Inhibitor Cocktail (Sigma‐Aldrich Chemical Co., Saint Louis, MO, USA); the mixture was then concentrated 100‐fold using an Amicon YM10 membrane (EMD Millipore, Billerica, MA, USA), dialyzed against distilled water and stored at −20°C until use.

### 2.5 Serum Samples

This is an exploratory study that used a total of 158 human serum samples to evaluate four types of antigenic preparations, composed of two panels of 79 samples each from Brazilian and Peruvian individuals. Each panel was composed of three subgroups as follows:

- *Positive group (G1)*: Comprised 20 samples from Brazilian (G1‐BR) and 20 serum samples of Peruvian (G1‐PE) individuals infected with *S. stercoralis* (presence of larvae in stool samples);
- *Negative group (G2)*: Comprised 39 samples from apparently healthy Brazilian (G2‐BR) and 39 samples from Peruvian (G2‐BR) individuals with negative diagnosis for intestinal parasitic infections;
- *Other parasitic infections group (G3)*: Comprised 20 samples from Brazilian (G3‐BR) and 20 samples from Peruvian (G3‐PE) individuals with a positive diagnosis of parasitic infection other than *S. stercoralis*. G3‐BR included infections by the parasites *Hymenolepis nana* (*n* = 5), hookworms (*n* = 3), *Schistosoma mansoni* (*n* = 7), *Ascaris lumbricoides* (*n* = 3), *Enterobius vermicularis* (*n* = 1) and *Giardia lamblia* (*n* = 1). G3‐PE included infections by the parasites *Hymenolepis nana* (*n* = 6), hookworms (*n* = 3), *Ascaris lumbricoides* (*n* = 5) and *Trichuris trichiura* (*n* = 6).

The presence or absence of intestinal parasites, including *S. stercoralis*, was determined by analysing a single stool sample using direct faecal smear examination, spontaneous sedimentation technique [18, 19] and coproculture in agar plate [20]. Diagnosis of tissue‐infecting parasites, including toxocariasis, cystic echinococcosis and cysticercosis, could not be performed due to study limitations.

### 2.6 Enzyme‐Linked Immunosorbent Assay for Detection of IgG Antibodies (ELISA‐IgG)

ELISA‐IgG was performed following the procedure described by Gomes et al. [12], with some modifications. The test was previously standardised by checkerboard titration using different concentrations of the antigenic preparations, different dilutions for serum samples and different dilutions of the secondary enzyme‐conjugated antibody. The following procedure describes the best conditions obtained with each one of the four different antigenic preparations. Briefly, Costar 96‐well flat bottom microtitration polystyrene plates (Sigma‐Aldrich Chemical Co., Saint Louis, MO, USA) were coated overnight at 4°C (1 μg/well) with each type of the four antigenic preparations diluted in carbonate–bicarbonate buffer (0.05 M, pH 9.6). The plates were washed three times for 5 min each with PBS containing 0.05% Tween‐20 (PBS‐T) and then blocked with 5% nonfat milk diluted in PBS‐T for 2 h at 37°C. After three washings with PBS‐T, Brazilian and Peruvian sera were diluted in blocking solution (1/200), added in duplicate to each well and incubated for 45 min at 37°C. After three washings with PBS‐T, peroxidase‐conjugated goat anti‐human IgG (Fc specific) antibody (Sigma‐Aldrich Chemical Co., St. Louis, MO, USA) was diluted (1/20,000) in blocking solution, added to each well, and incubated for 45 min at 37°C. After three washings with PBS‐T, the 3,3′,5,5‐tetramethylbenzidine (TMB) chromogen substrate solution (Thermo Fischer Scientific, Waltham, MA, USA) was added to each well, incubated for 7 min at 37°C and the enzymatic reaction was stopped with 2N sulphuric acid. Each plate was read at 450 nm using an ELISA reader (Thermo Fischer Scientific, Waltham, MA, USA) and the results were recorded as optical density (OD) values. All assays were monitored by including a positive (pool of serum samples from G1) and negative (pool of serum samples from G2) control sera as well as a blank (PBS instead of serum sample). An intra‐assay coefficient of variation (CV) of 10% or less was tolerated in the results of positive and negative control sera as an internal control in each assay [21].

### 2.7 Data Analysis

Statistical analysis was performed using the Graph Pad Prism version 5.0 (GraphPad Software, San Diego, CA). Descriptive statistics and D'Agostino‐Pearson normality test were performed on the ELISA results of both Brazilian and Peruvian subgroups of sera (G1, G2, G3). The unpaired *t*‐test was applied when both subgroups of sera passed the normality test; otherwise, the Mann–Whitney U test was used.

In order to evaluate the diagnostic accuracy of each antigen preparation, sera from G1 (cases) were used to calculate diagnostic sensitivity and sera from G2 (controls) were used to calculate diagnostic specificity following the receiver operating characteristic (ROC) curve analysis with a confidence interval (CI) of 95%, in which cut‐off values for 100% sensitivity or 100% specificity were calculated. Sera from G3 (other parasitic infections) were used to evaluate the cross‐reactivity rate using each cut‐off value obtained after ROC curve analysis. Other measures of diagnostic accuracy such as area under the ROC curve (AUC) and likelihood ratio (LR) were also calculated for each of the Brazilian and Peruvian serum groups [22].

After evaluating the diagnostic performance of the four antigenic extracts on serum panels from the two countries, an integrated evaluation was carried out combining the two aforementioned serum panels into one and determining which antigenic extract would be capable of detecting IgG antibodies in both countries with the best diagnostic performance.

### 2.8 Ethics approval

The study received approval of the Ethics Committee of the Hospital das Clínicas da Faculdade de Medicina da Universidade de São Paulo (CAAE 75472223.1.0000.0068). The life cycle maintenance of *S. venezuelensis* strain rats was conducted in accordance with animal ethics guidelines adopted by Comite de Etica em Experimentação Animal (CEUA) of the Instituto de Medicina Tropical da Faculdade de Medicina da Universidade de São Paulo (IMT/USP) (protocol n° 2291/2025).

## 3 Results

The reactivity of sera from the Brazilian and Peruvian G1, G2 and G3 against each of the four types of antigenic preparations evaluated by ELISA‐IgG is shown in Table 1. Serum samples from Brazilian subgroups G1 (*t* = 3.90; *p* = 0.0004) and G2 (*U* = 194; *p* < 0.0001) were more reactive than their Peruvian counterparts for the antigenic preparation S‐L3, with the exception of subgroup G3 where the results of Brazilian and Peruvian sera were no different (*U* = 136; *p* = 0.0859). Similarly, sera from Brazilian G1 (*U* = 37; *p* < 0.0001) and G2 (*U* = 557; *p* = 0.0420) were more reactive than their Peruvian counterparts for the antigenic preparation M‐L3, with the exception of G3 where the difference of the results was no different (*U* = 147; *p* = 0.1556). On the other hand, sera from Brazilian G1 were more reactive than Peruvian G1 (*U* = 111; *p* = 0.0167) for the antigenic preparation C‐L3, whereas the reactivity of sera from the Brazilian G2 (*U* = 637; *p* = 0.2171) and G3 (*U* = 188; *p* = 0.7557) subgroups was no different from their Peruvian counterparts. With regards to the antigenic preparation ES‐L3, sera from Brazilian subgroups G1 (*t* = 7.16; *p* < 0.0001), G2 (*U* = 4.00; *p* < 0.0001) and G3 (*U* = 47.0; *p* < 0.0001) were more reactive than their Peruvian counterparts.

**TABLE 1:** Characteristics of the ELISA‐IgG reactivity of sera from Brazilian (BR) and Peruvian (PE) individuals infected with *S. stercoralis* (G1), negative (G2) and individuals with other parasitic infections (G3) against the four types of antigenic preparations (S‐L3, M‐L3, C‐L3 and ES‐L3) from *S. venezuelensis* infective larvae.

Group of sera	Antigenic preparations							
	S‐L3		M‐L3		C‐L3		ES‐L3	
	BR	PE	BR	PE	BR	PE	BR	PE
Strongyloidiasis group—G1 (*n* = 20 each)								
Minimum	0.397	0.429	0.165	0.224	0.241	0.311	0.697	0.502
Median	1.340	0.812	2.050	0.450	0.602	0.972	2.680	0.857
Maximum	2.710	1.550	2.830	1.110	2.290	1.960	3.800	1.850
Mean	1.440	0.835	1.770	0.489	0.729	1.030	2.490	0.957
Standard deviation	0.622	0.299	0.882	0.216	0.505	0.389	0.881	0.376
Healthy group—G2 (*n* = 39 each)								
Minimum	0.073	0.051	0.056	0.059	0.057	0.066	0.130	0.062
Median	0.131	0.082	0.118	0.096	0.106	0.131	0.236	0.086
Maximum	0.402	0.253	0.387	0.325	0.358	0.381	0.643	0.145
Mean	0.162	0.089	0.137	0.105	0.139	0.160	0.278	0.091
Standard deviation	0.080	0.036	0.074	0.043	0.076	0.079	0.131	0.021
Other parasitic infections—G3 (*n* = 20 each)								
Minimum	0.059	0.056	0.061	0.060	0.055	0.053	0.201	0.063
Median	0.125	0.085	0.121	0.085	0.098	0.091	0.305	0.107
Maximum	0.610	0.408	0.275	0.232	0.541	0.723	1.090	0.570
Mean	0.200	0.134	0.131	0.103	0.125	0.144	0.395	0.160
Standard deviation	0.148	0.095	0.062	0.045	0.105	0.151	0.215	0.125

ROC curve analysis showed that the antigenic preparation ES‐L3 had an AUC of 1.0 (95% CI = 1.0–1.0; *p* < 0.0001) for both Brazilian and Peruvian sera followed by S‐L3 that had an AUC of 1.0 (95% CI = 0.99–1.0; *p* < 0.0001) for Brazilian sera and 1.0 (95% CI = 1.0–1.0; *p* < 0.0001) for Peruvian sera. In addition, the antigenic preparation C‐L3 had an AUC of 0.98 (95% CI = 0.96–1.00; *p* < 0.0001) for Brazilian sera and 1.0 (95% CI = 0.99–1.0; *p* < 0.0001) for Peruvian sera, whereas M‐L3 had an AUC of 0.98 (95% CI = 0.96–1.0; *p* < 0.0001) for Brazilian sera and 0.99 (95% CI = 0.98–1.0; *p* < 0.0001) for Peruvian sera.

When the cut‐off value was chosen to achieve 100% sensitivity (when there is a need for a screening test for epidemiological surveys), the antigenic preparation ES‐L3 achieved a specificity of 100% for both Brazilian sera (cut‐off = 0.670) and Peruvian sera (cut‐off = 0.324), followed by S‐L3 which achieved a specificity of 100% for Peruvian sera (cut‐off = 0.341) and 97.4% for Brazilian sera (cut‐off = 0.367).

In addition, the antigenic preparation C‐L3 achieved a specificity of 97.4% for Peruvian sera (cut‐off = 0.302) and 84.6% for Brazilian sera (cut‐off = 0.230); M‐L3 achieved a specificity of 97.4% for Peruvian sera (cut‐off = 0.196) and 71.8% for Brazilian sera (cut‐off = 0.161). Under these conditions, the occurrence of cross‐reactivity in samples from individuals with other parasitic infections (G3), C‐L3 achieved the lowest value of cross‐reactivity (5%—1/20 in each country) in one sample of a Brazilian patient with *S. mansoni* and in one Peruvian patient with *A. lumbricoides*. Although ES‐L3 achieved 100% of both sensitivity and specificity, it had the occurrence of cross‐reactivity in one Brazilian patient infected with hookworm and in two Peruvian patients infected with *A. lumbricoides*. In addition, S‐L3 had the occurrence of cross‐reactivity in two Brazilian patients infected with *H. nana* and *S. mansoni* and in one Peruvian patient infected with *A. lumbricoides*, and M‐L3 had the occurrence of cross‐reactivity in six Brazilian patients infected with *A. lumbricoides, E. vermicularis*, hookworm (*n* = 2), *H. nana, S. mansoni* and in two Peruvian patients infected with *A. lumbricoides*.

When the cut‐off value was chosen to achieve 100% specificity (when there is a need for a confirmatory test), the antigenic preparation S‐L3 (cut‐off = 0.516) and M‐L3 (cut‐off = 0.395) achieved sensitivities of 95% for Brazilian sera. In addition, the antigenic preparation M‐L3 achieved specificities of 95% (cut‐off = 0.395) for Brazilian sera and of 80% (cut‐off = 0.337) for Peruvian sera, whereas C‐L3 achieved specificities of 75% (cut‐off = 0.359) for Brazilian sera and of 95% (cut‐off = 0.401) for Peruvian sera. With regard to the cross‐reactivity in group G3, M‐L3 has no occurrence of cross‐reactivity in serum samples from both countries, whereas S‐L3, C‐L3 and ES‐L3 had just one sample positive in serum samples from both countries.

These results show that all four antigenic preparations performed better on the Peruvian serum panel than on the Brazilian counterparts despite the high reactivity observed in the latter group (Table 2).

**TABLE 2:** Diagnostic parameters of antigenic extracts from L3 of *S. venezuelensis* (S‐L3, M‐L3, C‐L3 and ES‐L3) obtained from sera from Brazilian (BR) and Peruvian (PE).

Antigenic extracts		Cut‐off	LR	Sensitivity (95% CI)	Specificity (95% CI)
S‐L3	BR	> 0.367	39.0	100% (83.2%–100.0%)	97.4% (86.5%–99.9%)
		> 0.516	—	95% (75.1%–99.9%)	100% (91.0%–100.0%)
	PE	> 0.341	—	100.0% (83.2%–100.0%)	100.0% (91.0%–100.0%)
M‐L3	BR	> 0.161	3.5	100.0% (83.2%–100.0%)	71.8% (55.1%–85.0%)
		> 0.395	—	95.0% (75.1%–99.9%)	100.0% (91.0%–100.0%)
	PE	> 0.196	39.0	100.0% (83.2%–100.0%)	97.4% (86.5%–99.9%)
		> 0.337	—	80.0% (56.3%–94.3%)	100.0% (91.0%–100.0%)
C‐L3	BR	> 0.230	14.2	100.0% (83.2%–100.0%)	84.6% (69.5%–94.1%)
		> 0.359	—	75% (50.9%–91.3%)	100.0% (91.0%–100.0%)
	PE	> 0.302	39.0	100.0% (83.2%–100.0%)	97.4% (86.5%–99.9%)
		> 0.401	—	95.0% (75.1%–99.9%)	100.0% (91.0%–100.0%)
ES‐L3	BR	> 0.670	—	100.0% (83.2%–100.0%)	100.0% (91.0%–100.0%)
	PE	> 0.324	—	100.0% (83.2%–100.0%)	100.0% (91.0%–100.0%)

Given that some of the antigenic preparations have shown excellent performance in detecting anti‐*Strongyloides* IgG antibodies in serum panels from both countries, new cut‐off values were determined following the ROC curve analysis for each antigenic preparation applicable to both countries (Figure 1). In this new situation, the ROC curve analysis showed that the antigenic preparation S‐L3 had an AUC of 1.0 (95% CI = 1.0–1.0; *p* < 0.0001) followed by ES‐L3 that had an AUC of 1.0 (95% CI = 0.99–1.0; *p* < 0.0001). In addition, C‐L3 had an AUC of 0.99 (95% CI = 0.98 to 1.00; *p* < 0.0001), whereas M‐L3 had an AUC of 0.99 (95% CI = 0.98–1.00; *p* < 0.0001). For a cut‐off to achieve 100% sensitivity, the antigenic preparation S‐L3 showed a specificity of 98.7% (cut‐off = 0.367) followed by ES‐L3 that showed a specificity of 96.1% (cut‐off = 0.500); C‐L3 achieved a specificity of 84.6% (cut‐off = 0.239) and then M‐L3 that achieved a specificity of 83.3% (cut‐off = 0.163). Occurrence of cross‐reactivity was high in M‐L3 and ES‐L3 where 6/20 (30%) samples reacted positively with these two mentioned antigenic preparations, followed by S‐L3 that had a cross‐reactivity of 15% (3/20) and C‐L3 that achieved the lowest cross‐reactivity of 5% (1/20). For a situation of 100% specificity, S‐L3 achieved a sensitivity of 97.5% (cut‐off = 0.416) followed by ES‐L3 with a sensitivity of 90% (cut‐off = 0.644); C‐L3 achieved a sensitivity of 82.5% (cut‐off = 0.387), and then M‐L3 that achieved a sensitivity of 80% (cut‐off = 0.389). Under these conditions, the cross‐reactivity was totally eliminated in ES‐L3 and M‐L3, whereas one sample from G3 was positive with C‐L3 and two samples were positive with S‐L3.

**FIGURE 1:**
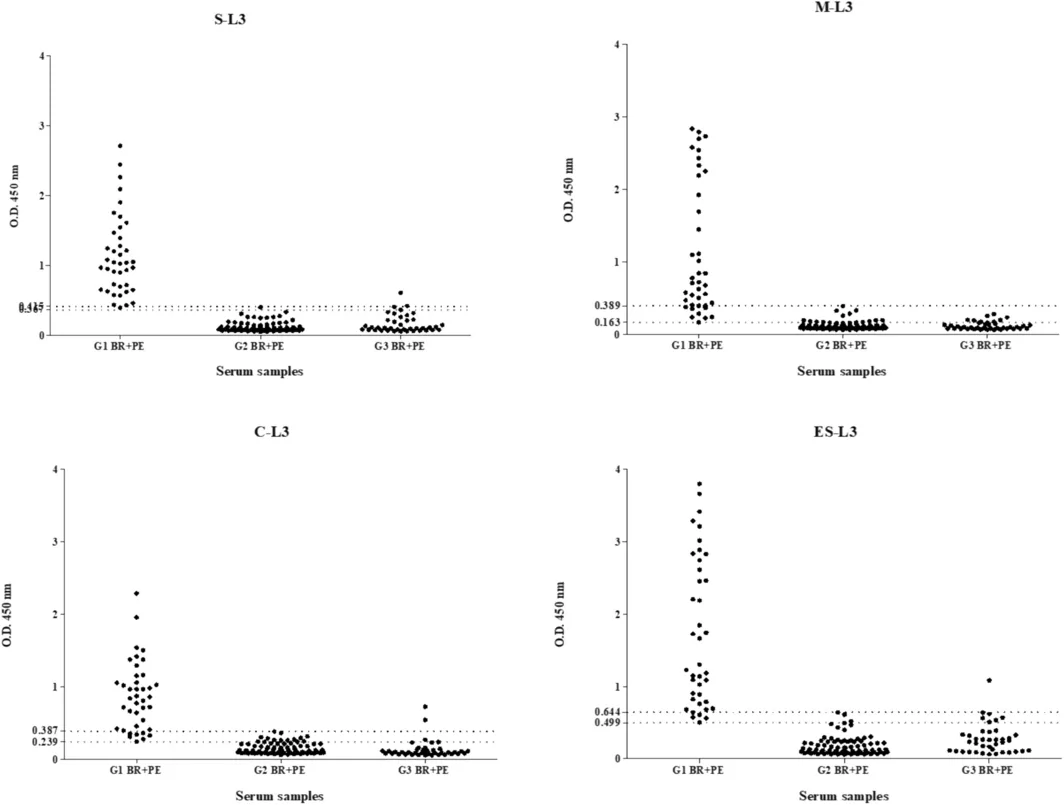
ELISA‐IgG reactivity (450 nm) of sera from Brazilian (BR) and Peruvian (PE) individuals infected with *S. stercoralis* (G1). negative (G2) and with other parasitic infections (G3) from Brazil (BR) and Peru (PE) against the four types of antigenic preparations (S‐L3, M‐L3, C‐L3, and ES‐L3) from *S. venezuelensis* infective larvae. The red line indicates the cut‐off value with 100% sensitivity and the black line indicates the cut‐off value with 100% specificity for each antigen preparation.

## 4 Discussion

Human strongyloidiasis remains an underdiagnosed and underestimated parasitic infection and represents a relevant public health issue worldwide. Recently, the World Health Organization included the control of strongyloidiasis among the targets for 2030 [23], reinforcing the need for reliable diagnostic tools applicable both to clinical practice and epidemiological surveys [6]. Although antibody detection has limitations for assessment of cure, ELISA‐based assays targeting anti‐*Strongyloides* IgG antibodies remain preferable to parasitological methods in both clinical and epidemiological settings due to their high sensitivity, reproducibility and suitability for high‐throughput analysis.

In this context, the establishment of appropriate cut‐off values is critical for the accurate interpretation of serological results [22, 24]. In the present study, all four antigenic preparations derived from *S. venezuelensis* iL3 showed AUC values above 0.90, indicating excellent diagnostic performance for the detection of anti‐*Strongyloides* IgG antibodies.

When analysed separately in Brazilian and Peruvian serum samples, all antigenic preparations exhibited good overall performance, with sensitivities ranging from 75% to 100% and specificities from 71.8% to 100% in Brazilian sera, and sensitivities from 80% to 100% and specificities from 97.4% to 100% in Peruvian sera. These findings are consistent with those reported by Kalantari et al. [10], who suggested that *S. venezuelensis* antigens may have greater diagnostic value than those derived from *S. stercoralis*. The use of antigens from non‐human‐infecting *Strongyloides* species also offers important practical advantages, such as greater ease of laboratory maintenance and continuous antigen availability [9, 25].

Despite the higher overall absorbance values observed in Brazilian sera, diagnostic performance was superior in the Peruvian population. This apparent discrepancy highlights that increased antibody reactivity does not necessarily translate into improved diagnostic accuracy. Differences in absorbance values between Brazilian and Peruvian samples may reflect population characteristics, infection intensity or duration, co‐infections, sample handling, assay variation, as well as potential differences among circulating *Strongyloides* strains. Recent studies have identified distinct *S. stercoralis* haplotypes in different geographic regions, suggesting the existence of genetic diversity that could contribute to antigenic variation and, consequently, differences in host immune recognition [26, 27, 28].

Compared with commercial ELISA kits evaluated in non‐endemic settings, which have frequently shown lower sensitivity and high cross‐reactivity [29], the antigenic preparations evaluated in this study demonstrated better diagnostic performance, particularly in sera from endemic populations. These findings highlight the importance of validating serological assays in endemic contexts, where exposure to other parasitic infections may increase the potential for cross‐reactivity and consequently affect assay specificity.

To establish standardised diagnostic criteria that could be applicable across different endemic regions, Brazilian and Peruvian serum samples were combined to derive unified cut‐off values. Under this integrated approach, S‐L3 showed the best overall diagnostic performance, with 100% sensitivity and 98.7% specificity. Notably, when the cut‐off was adjusted to prioritise specificity, S‐L3 retained high sensitivity while further reducing the potential for cross‐reactive reactions. This performance supports the potential of S‐L3 as a promising antigen for the development of standardised, high‐performance serological assays for the diagnosis of human strongyloidiasis in various endemic settings. Nevertheless, its applicability requires confirmation through prospective validation using larger and geographically diverse cohorts.

Several limitations of the study should be considered. First, the relatively small number of samples evaluated from each country may limit the precision and robustness of the estimated diagnostic parameters. Secondly, because the study relied on archived samples with limited clinical and epidemiological information, the biological interpretation of the differences observed between the Brazilian and Peruvian panels is constrained. Third, classification of groups based on parasitological examination of a single stool sample may have affected the estimated diagnostic performance of the antigenic preparations. Fourth, the absence of samples from individuals with tissue‐invasive helminth infections in the panels from both countries limited the assessment of potential cross‐reactivity and may have led to an overestimation of assay specificity. Finally, the cut‐off derived from the combined Brazilian and Peruvian datasets should be interpreted with caution. Ideally, cross‐validation between the countries would be performed by establishing the threshold in Brazil and evaluating it in Peru, and vice versa. These limitations point to clear priorities for future research, including the evaluation of larger and geographically diverse cohorts from both countries, together with parasitological diagnosis based on the examination of at least three stool samples. Prospective studies incorporating detailed clinical and epidemiological characterisation, as well as control groups with other helminth infections, will be important to validate the diagnostic performance of these heterologous antigenic preparations.

## 5 Conclusion

The recognition of distinct antigenic preparations of *S. venezuelensis* L3 by antibodies present in serum samples from Brazil and Peru opens new perspectives for the serological diagnosis of *S. stercoralis* infection. Collectively, these results indicate that heterologous antigenic preparations from *S. venezuelensis* may represent a useful alternative for the serodiagnosis of *S. stercoralis* infection in endemic settings such as Brazil and Peru.

## Author Contributions

W.H.R. contributed to the study design, performed diagnostic tests and wrote and reviewed the manuscript. M.A.C. contributed to the study design, prepared antigenic extracts and revised the manuscript. D.M.C.L.M. prepared antigenic extracts and performed diagnostic tests. M.C. and M.A.T. performed diagnostic tests and reviewed the manuscript. S.V.L. and R.C.B. contributed to the manuscript design and revision. F.M.P. contributed to the study design, provided serum samples, secured funding and wrote and reviewed the manuscript. All authors reviewed the manuscript.

## Funding

This research was supported by Fundação de Amparo à Pesquisa do Estado de São Paulo (FAPESP 2022/02401‐1 and 2023/07417‐6) and Conselho Nacional de Desenvolvimento Científico e Tecnológico (CNPq, 310071/2022‐4) of Brazil. The Article Processing Charge for the publication of this research was funded by the Coordenação de Aperfeiçoamento de Pessoal de Nível Superior ‐ Brasil (CAPES) (ROR identifier: 00x0ma614).

## Conflicts of Interest

The authors declare no conflicts of interest.

## Data Availability

The data that support the findings of this study are available from the corresponding author upon reasonable request.
